# Habitat Suitability Assessment of Milu (*Elaphurus davidianus*) in Coastal Wetlands of Jiangsu Province Based on Species Distribution Models

**DOI:** 10.3390/ani16121871

**Published:** 2026-06-17

**Authors:** Fan Sheng, Xinyu Shen, Liangsong Xie, Bin Liu, Jian Huang, Geng Huang, Ranxing Cao, Yifei Jia, Yan Zhou

**Affiliations:** 1 Co-Innovation Center for Sustainable Forestry in Southern China, College of Life Sciences, Nanjing Forestry University, Nanjing 210037, China; refan03@163.com (F.S.); 19850530908@163.com (X.S.); 15970813324@163.com (L.X.); 2 Jiangsu Dafeng Milu National Nature Reserve, Yancheng 224136, China; 15861952240@163.com; 3 Shenzhen Mangrove Wetlands Conservation Foundation, Shenzhen 518034, China; huangjian@mcf.org.cn; 4 School of Ecology and Nature Conservation, Beijing Forestry University, Beijing 100083, China; ye00ow@bjfu.edu.cn (G.H.); jiayifei@bjfu.edu.cn (Y.J.); 5 Centre for East Asian-Australasian Flyway Studies, Beijing Forestry University, Beijing 100083, China; 6 College of Life Sciences, Beijing Normal University, Beijing 100875, China; cranxing@mail.bnu.edu.cn

**Keywords:** Milu, coastal wetlands, habitat suitability modeling, geographical detector, climate change

## Abstract

Under global climate change, ungulate distributions are generally shifting poleward. However, the dispersal pathways, dynamics of suitable habitat, and environmental drivers of the expanding Milu population in the intensively used coastal wetlands of Jiangsu Province remain poorly understood. To support conservation and management, this study used field occurrence data and environmental variables to predict potentially suitable habitat for Milu under current and future climate scenarios. The Biomod2 ensemble modeling framework was applied to assess spatial changes in habitat suitability, and Geographical Detector was used to identify key environmental drivers. Current potentially suitable habitat showed a belt-like pattern along the coast, with the high suitability area covering 0.035 × 10^4^ km^2^. Under future climate scenarios, potentially suitable habitat for Milu is projected to expand in the central and northern coastal areas of Jiangsu, with a substantial increase in the predicted total suitable habitat area. Dis_coastline, BIO14, BIO4, and Pop_density were identified as key factors influencing the distribution of potential suitable habitat for Milu, among which BIO4 and BIO14 were the principal climatic drivers affecting the northward shift in future suitable areas. These results suggest that Milu habitat suitability is jointly shaped by coastline proximity, temperature and water-availability conditions, and population density. Conservation should prioritize the protection of highly suitable habitats, improve patch connectivity, reduce human disturbance, and strengthen wetland protection and vegetation restoration.

## Simple Summary

Milu (*Elaphurus davidianus*) is a globally endangered wetland ungulate, and the coastal wetlands of Jiangsu Province support the largest wild population of this species worldwide. Under climate change and increasing human disturbance, identifying shifts in suitable habitat for Milu is critical for conservation planning. Using GPS tracking data and environmental variables, we modeled current and future habitat suitability in the coastal wetlands of Jiangsu Province using the Biomod2 ensemble modeling framework and examined the driving factors using the optimal parameter-based Geographical Detector (OPGD). The results indicated that currently suitable habitats are concentrated along the coastline and are expected to expand further in the central and northern coastal areas of Jiangsu Province. Distance to the coastline, precipitation of the driest month (BIO14), temperature seasonality (BIO4), and population density jointly affected habitat distribution. These findings provide scientific guidance for population management, habitat conservation, ecological corridor construction, and the mitigation of human disturbance in Jiangsu’s coastal wetlands.

## 1. Introduction

Under ongoing global climate change and increasing habitat fragmentation, the distribution patterns and habitat-use strategies of ungulates have changed markedly [1,2]. As sensitive indicator species of environmental change, ungulates respond to climatic fluctuations by shifting their geographical ranges and modifying habitat use [3]. Studies have shown that ungulate habitats generally tend to shift northward and toward higher elevations [4,5], as observed in species such as *Equus kiang*, *Procapra picticaudata*, *Bos mutus*, *Elaphodus cephalophus*, and *Odocoileus virginianus* [6,7,8,9]. In contrast, historically occupied habitats at lower latitudes and elevations may experience declines in area and habitat quality due to extreme climatic events and vegetation changes [10,11], thereby complicating future habitat identification and protected-area planning.

As a key species in wetland ecosystems, Milu is a rare, large ungulate endemic to China. It is listed as a Class I nationally protected wild animal in China (2021), was classified as Extinct in the Wild (EW) by the International Union for Conservation of Nature (IUCN) in 2016, and is listed as Critically Endangered (CR) in the China Red List [12]. The recovery of Milu populations represents an important example in the history of global biodiversity conservation. However, within China, the recovery process differs among regions. The population in Jiangsu Dafeng Reserve has recovered most dramatically, exceeding 8000 individuals [13], far higher than the rewilded populations in Dongting Lake, approximately 209 individuals, and Shishou, Hubei Province, with more than 1200 individuals [14,15]. Owing to rapid population recovery, some individuals in Dafeng have begun to forage outside the reserve, entering surrounding woodlands, farmlands, and aquaculture ponds, thereby intensifying spatial competition between humans and Milu [16,17]. Currently, some wild individuals from Dafeng have dispersed to coastal mudflats in Rudong, Nantong [18], forming a distribution belt along the coastline with Dafeng Reserve as the core area. The coastal region of Jiangsu is an important distribution area for the largest Milu population in China and worldwide [19], where the population continues to expand despite intense human activities. Under global warming, Milu populations generally show a northward shift; however, habitat fragmentation has led to a scattered distribution of suitable habitat patches [20]. Nevertheless, the dispersal pathways, habitat dynamics, and environmental driving mechanisms of Milu in coastal Jiangsu remain insufficiently studied. Based on this background, we proposed the following ecological hypotheses: (1) current habitat suitability for Milu in coastal Jiangsu is jointly influenced by coastal proximity, climatic conditions, and anthropogenic disturbance, with suitable habitats mainly occurring in coastal areas characterized by favorable climatic conditions and relatively low human disturbance; (2) under future climate scenarios, the spatial pattern of potentially suitable habitat may change and shift toward northern coastal Jiangsu; and (3) climatic factors may be important drivers of future changes in potentially suitable habitat for Milu.

Therefore, this study focuses on the Milu population distributed in coastal Jiangsu. Using field occurrence records obtained from GPS collar tracking and environmental variables, we constructed a habitat suitability model to test these ecological hypotheses. Specifically, this study aimed to (1) analyze the distribution pattern of Milu in coastal Jiangsu and identify the main environmental factors influencing its distribution; (2) explore the spatiotemporal dynamics of potentially suitable habitat under different future climate scenarios; and (3) identify the key drivers of future changes in potentially suitable habitat, thereby providing a scientific basis for the long-term conservation of Milu populations and the mitigation of human-deer conflicts.

## 2. Materials and Methods

### 2.1. Study Area

**Figure 1:**
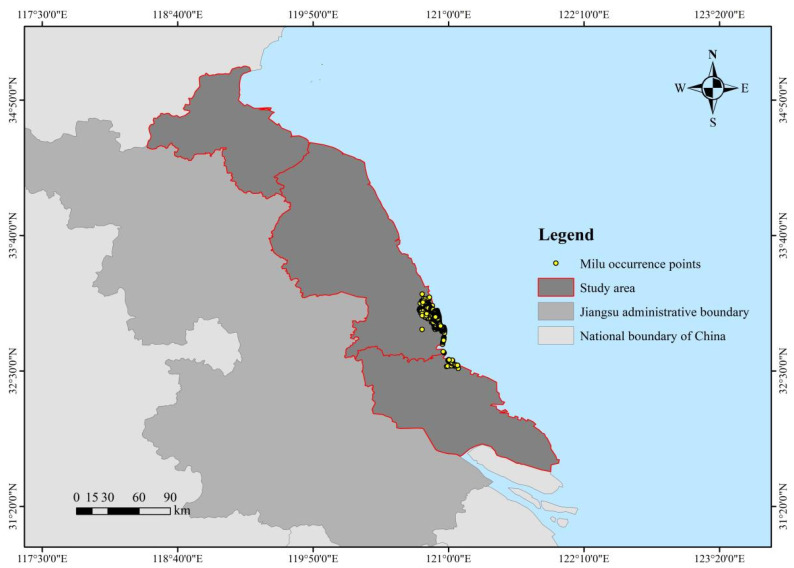
Study area and occurrence points of Milu in coastal Jiangsu Province, China. Yellow dots represent GPS-based Milu occurrence points; the red outline indicates the study area, including the coastal cities of Lianyungang, Yancheng, and Nantong; dark grey shading represents the Jiangsu administrative boundary; light grey shading represents areas outside Jiangsu Province; and light blue indicates the sea area.

The study area (Figure 1) comprised three coastal cities in Jiangsu Province: Lianyungang, Yancheng, and Nantong. It is located between 31.41 and 35.08° N and 118.24–121.54° E. The region has a northern subtropical monsoon climate, with an annual mean temperature of approximately 14–16 °C and annual precipitation ranging from 800 to 1100 mm [21,22]. The terrain is generally flat and low lying, with most areas at elevations of 3–5 m [23]. The Milu release site was located near the estuary of the Liangduo River in Dongtai, Yancheng, Jiangsu Province. This area is characterized by flat terrain and abundant water resources, making it a suitable habitat for wild Milu populations.

### 2.2. Prediction of Potential Habitats of Milu Based on the Biomod2 Model

Species distribution models (SDMs) are important tools in modern ecology for predicting species’ spatial distributions [24,25]. By integrating species occurrence records with environmental variables, SDMs can characterize ecological niche features and further predict potential habitat distributions within specific spatial and temporal ranges [26]. With advances in ecological niche modeling, the bioclimatic envelope algorithm (BIOCLIM) [27], generalized linear model (GLM) [28], maximum entropy model (MaxEnt) [29], random forest model (RF) [30], and other approaches have been widely used for species distribution prediction. Because predictions from individual algorithms are often uncertain, the Biomod2 ensemble modeling framework has become widely used in potential habitat studies [31]. By integrating multiple algorithms, such as GLM, MaxEnt, and RF, ensemble modeling can improve output reliability and provide more stable predictions of potentially suitable habitat for Milu in the complex environment of Jiangsu’s coastal wetlands.

#### 2.2.1. Milu Occurrence Data

In this study, nine Milu individuals were fitted with GPS tracking collars (Druid Technology, Chengdu, China) for wildlife monitoring. All individuals were released in April 2023 near the estuary of the Liangduo River in Dongtai, Jiangsu Province. From 5 April 2023 to 4 April 2025, a total of 96,110 occurrence records were collected from the nine individuals. To reduce the influence of spatial clustering on SDM predictions, the original occurrence data were filtered to remove redundant records before modeling [32]. ENMTools [33] was used for spatial filtering, with only one record retained in each grid cell. Ultimately, 7688 valid occurrence records were retained for subsequent analysis.

#### 2.2.2. Acquisition and Processing of Environmental Variables

Future climate data were obtained from the BCC-CSM2-MR model [34], which has been shown to perform well over China, within the framework of the Coupled Model Intercomparison Project Phase 6 (CMIP6) coordinated by the World Climate Research Programme (WCRP) [35]. To compare potential habitat responses of Milu under different climate change intensities, three scenarios were selected: SSP1-2.6 (SSP126), SSP2-4.5 (SSP245), and SSP3-7.0 (SSP370), representing low-, medium-, and relatively high-emission pathways, respectively [36,37]. Raster layers of the 19 bioclimatic variables were downloaded for the current period (1970–2000) and future periods (2041–2060 and 2061–2080) were downloaded from the WorldClim 2.1 database at a spatial resolution of 30 as (approximately 1 km).

In addition to climatic variables, 29 environmental variables related to geography, habitat, and human disturbance were incorporated to construct the habitat suitability model for Milu and to identify key factors influencing habitat suitability (Table 1) to construct the habitat suitability model for Milu and identify key factors influencing habitat suitability. Elevation data were obtained from the Copernicus Digital Elevation Model provided by the Copernicus Data Space Ecosystem (https://dataspace.copernicus.eu/, accessed on 14 June 2026), with a spatial resolution of 30 m. Coastline data were derived from the 1:1,000,000 public version of the basic geographic information dataset released in 2021 by the Catalogue Service for Geographic Information Resources of China (https://mulu.tianditu.gov.cn/), and the Euclidean distance from each raster cell to the nearest coastline was calculated in ArcGIS 10.8. River data were obtained from OpenStreetMap (https://www.openstreetmap.org/), and the distance to the nearest river was calculated using Euclidean distance in ArcGIS 10.8. Vegetation was represented by the annual normalized difference vegetation index (NDVI) dataset for China, obtained from the Resource and Environmental Science Data Registration and Publishing System (https://www.resdc.cn), with a spatial resolution of 1 km. Land-use data were obtained from the annual China land cover dataset developed by Wuhan University [38] based on the Google Earth Engine (GEE) platform, with a spatial resolution of 30 m. The Human Footprint Index (HFI) was obtained from the Socioeconomic Data and Applications Center (SEDAC), with a spatial resolution of approximately 1 km. Nighttime light data were obtained from the Harvard Dataverse platform, with a spatial resolution of 1 km. Vector data for roads and settlements were also obtained from the 1:1,000,000 public version of the basic geographic information dataset released in 2021 by the Catalogue Service for Geographic Information Resources of China. The Euclidean distance from each raster cell to the nearest road and settlement was calculated in ArcGIS 10.8, and the corresponding distance raster layers were generated. Population density data were downloaded from WorldPop (https://hub.worldpop.org), with a spatial resolution of 1 km.

In ArcGIS 10.8, the vector boundary of the three coastal cities of Jiangsu was used as the study area extent. The administrative boundary vector data were sourced from the National Geomatics Center of China (NGCC). All raster layers were resampled to a cell size of 100 m × 100 m, with their spatial resolution, numbers of rows and columns, spatial extent, and coordinate system standardized to WGS 1984 [39,40,41], ensuring consistency among raster inputs for the Biomod2 model. Finally, the Raster to ASCII tool was used to batch-convert the environmental raster data into ASC-format files.

Before constructing the Biomod2 ensemble model, multicollinearity among environmental variables was assessed [42,43]. Correlation screening was performed in R, and environmental variables with an absolute correlation coefficient greater than 0.8 were removed [44,45]. Variance inflation factor (VIF) analysis [46,47] was then conducted, and variables with VIF values greater than 10 were excluded [48,49]. Only variables retained by both screening procedures were included in the final set of environmental variables for constructing the Biomod2 model.

#### 2.2.3. Ensemble Model Construction and Evaluation

In this study, the biomod2 package version 3.5.1 in R 4.4.2 was used to predict current and future potentially suitable habitat for Milu in the coastal wetlands of Jiangsu. A total of 12 algorithms were used for single-model construction, including the generalized linear model (GLM), generalized additive model (GAM), multivariate adaptive regression splines (MARS), artificial neural network (ANN), random forest (RF), generalized boosted regression model (GBM), extreme gradient boosting (XGBOOST), maximum entropy model (MaxEnt), maximum entropy network (MAXNET), classification tree analysis (CTA), flexible discriminant analysis (FDA), and surface range envelope (SRE) [50]. These algorithms represent different modeling approaches, including regression, classification, machine learning, boosting, and maximum entropy methods, allowing the effects of different model assumptions on prediction outcomes to be compared under identical data conditions. The internal parameters of each individual model were set to the default settings of the biomod2 package and its underlying R functions to ensure workflow consistency, reproducibility, and comparability among algorithms. According to the design of this study, pseudo-absence generation, training/testing partitioning, the number of repeated runs, model evaluation metrics, and ensemble modeling methods were manually specified. Specifically, three sets of 8000 randomly generated pseudo-absence points were used. In each run, 75% of the occurrence records of Milu were used as the training set, and the remaining 25% were used as the testing set for validation [51,52]. Model calibration was repeated 10 times for each set of pseudo-absence points. The True Skill Statistic (TSS) and the Area Under the Receiver Operating Characteristic Curve (AUC) were used to evaluate model performance [53]. Finally, individual models with both TSS and AUC values greater than 0.8 were selected to construct the ensemble model using the ensemble modeling weighted mean method (EMwmean) [54], which assigns weights to individual models based on their evaluation performance. As a result, models with higher predictive accuracy make greater contributions to the ensemble prediction, thereby reducing the influence of poorly performing models on the final output. This approach reduced the uncertainty associated with individual models through multi-algorithm ensemble modeling and improved the stability and relative reliability of habitat suitability predictions for Milu in coastal Jiangsu. For future scenario projections, the climate variables under the SSP126, SSP245, and SSP370 scenarios for the 2050s and 2070s were replaced with their corresponding future values, while all non-climatic variables were held constant at their current values and used as baseline background conditions in model projections to assess the effects of future climate change on the spatial pattern of potential suitable habitat for Milu [55,56,57].

Model accuracy was evaluated using AUC and TSS. AUC ranges from 0 to 1, with values closer to 1 indicating better model performance [58]. Generally, an AUC value of 0.5–0.6 indicates poor predictive performance, 0.6–0.7 fair performance, 0.7–0.8 good performance, 0.8–0.9 very good performance, and value of 0.9–1.0 indicate excellent predictive performance. TSS ranges from −1 to 1. Negative values generally indicate poor performance; a TSS value below 0.4 indicates model prediction failure, 0.4–0.5 fair performance, 0.5–0.7 good performance, 0.7–0.8 very good performance, and 0.8–1.0 indicates excellent predictive performance [59].

For the classification of potential habitat suitability classes, the prediction results of the Biomod2 ensemble model were imported into ArcGIS 10.8 and normalized using the Raster Calculator. The habitat suitability probability of each raster cell ranged from 0 to 1, with higher values indicating greater suitability for Milu. A value of 0 indicated that the area was unsuitable, whereas a value of 1 indicated the highest probability of suitability within the study area. The predicted raster results were then classified into four suitability classes based on probability values using the natural breaks classification method [60,61].

### 2.3. Analysis of Driving Factors of Potential Habitats of Milu Based on the OPGD Model

The Geographical Detector is a statistical method used to detect spatially stratified heterogeneity and can effectively reveal the effects of explanatory factors on spatial patterns and their underlying mechanisms [62,63]. The optimal-parameter-based Geographical Detector (OPGD) is a spatial heterogeneity detection method derived from the traditional Geographical Detector. It uses the GD package version 10.8 in R version 4.4.2 to perform optimal discretization of continuous variables [64]. Specifically, continuous variables are classified into 3–8 categories using different discretization methods, including the equal interval method, natural breaks method, quantile method, geometric interval method, and standard deviation method. The q-values under different combinations of discretization methods and classification numbers are then calculated [65]. Finally, the discretization method and classification level with the maximum q-value are selected as the optimal parameter combination for each factor. The q-value was used to quantify the explanatory power of each factor, and was calculated Equation (1) as follows:

(1) $$q=1\;-\;\frac{\sum_{h=1}^{L}N_{h}\sigma_{h}^{2}}{N\sigma^{2}}$$

where q represents the explanatory power of explanatory variable X for the dependent variable Y and ranges from 0 to 1. A higher q-value indicates stronger explanatory power of X for Y, whereas a lower value indicates weaker explanatory power. L represents the number of strata of the dependent variable Y or explanatory variable X; N_h_ and N represent the number of units in stratum h and in the entire study area, respectively; and $\sigma_{h}^{2}$ and $\sigma^{2}$ represent the variance of Y in stratum h and in the entire study area, respectively [66].

In this study, the factor detector was first used to verify the key environmental factors selected by the Biomod2 model. The environmental factors that passed the verification were then analyzed using interaction detection to identify the main driving factors affecting the potential habitat distribution of Milu. The selected environmental factors were used as explanatory variables, while the predicted habitat suitability of Milu in the study area was used as the dependent variable. This analysis was designed to examine spatial heterogeneity in Milu distribution and its drivers. The model assumes that if environmental factor X has a significant driving effect on the distribution of Milu, then X and the dependent variable Y should show significant spatial similarity. Unlike the variable importance and response curves derived from the Biomod2 model, OPGD focuses on explaining the spatial heterogeneity of potential suitable areas and the interactions among multiple factors.

## 3. Results

### 3.1. Current Potential Habitats and Dominant Factors of Milu

The performance of the 12 species distribution models was comprehensively compared using the evaluation metrics. An ensemble model was then constructed using the EMwmean method and the 10 selected single models, including CTA, FDA, GAM, GBM, GLM, MARS, MAXENT, MAXNET, RF, and XGBOOST. After processing the prediction results, habitat suitability was classified into four categories using the natural breaks method: high suitability area (0.653, 1], moderate suitability area (0.319, 0.653], low suitability area (0.124, 0.319], and unsuitable area [0, 0.124].

**Figure 2:**
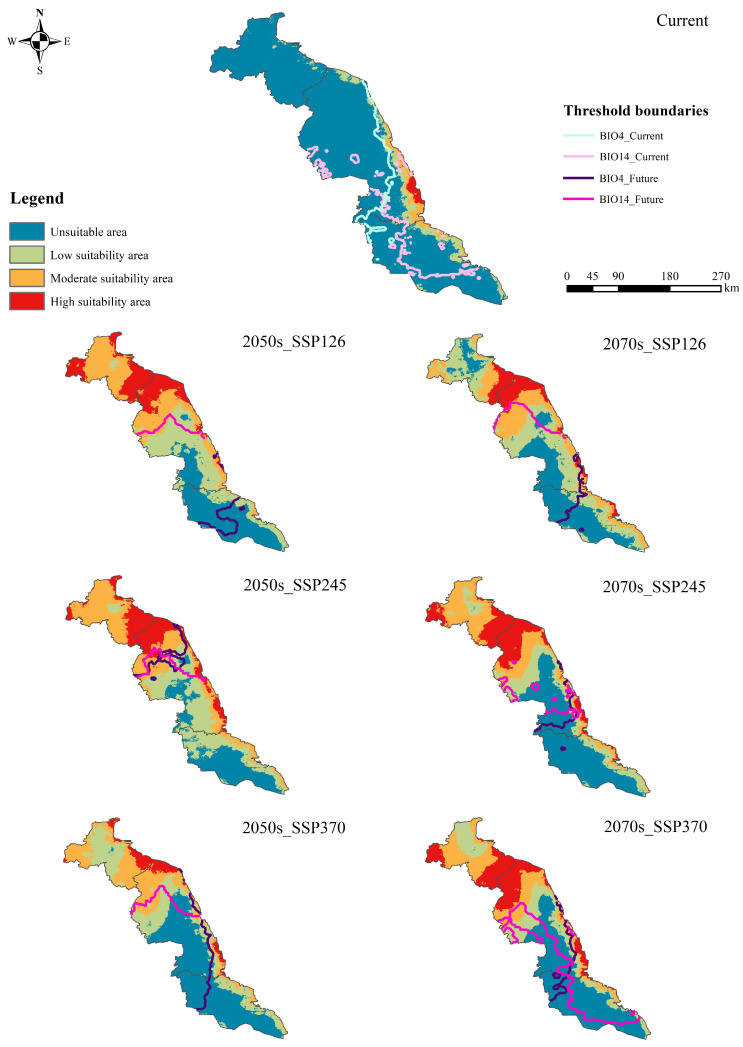
Potential suitable habitat of Milu under current and future climate scenarios and changes in BIO4 and BIO14 threshold lines.

Using ArcGIS 10.8, the land area of the three coastal cities in Jiangsu Province was calculated as approximately 31,520 km^2^ under the unified land-area statistical criteria adopted in this study. The total area of potentially suitable habitat for Milu was 0.370 × 10^4^ km^2^ (Table 2), and this habitat was mainly distributed along the coastal zone, showing a distinct belt-like pattern and extending northward and southward along the coastline. Unsuitable areas accounted for approximately 88.3% of the total area and were mainly located in inland areas far from the coastline and in peripheral offshore areas. Overall, currently suitable habitat for Milu was characterized by coastal aggregation, a gradual decrease in suitability toward inland areas, and localized concentration in some areas (Figure 2).

**Figure 3:**
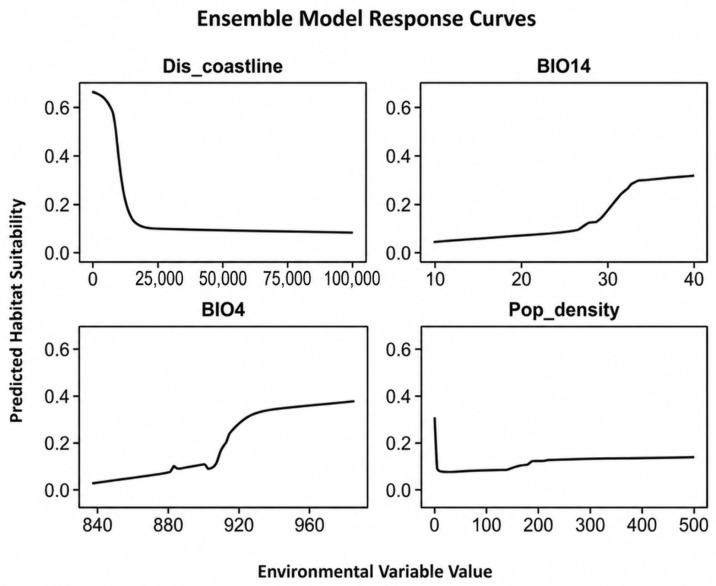
Response curves of key environmental variables affecting the potential habitat suitability of Milu. The x-axis represents the values of environmental variables, and the y-axis represents the habitat suitability probability predicted by the ensemble model. The four variables shown are distance to coastline (Dis_coastline), precipitation of the driest month (BIO14), temperature seasonality (BIO4), and population density (Pop_density).

Based on correlation analysis and VIF screening, 14 environmental factors were retained: BIO1, BIO4, BIO12, BIO14, Altitude, Dis_coastline, Dis_river, Dis_road, Dis_settlement, HFI, LC, NDVI, NTL, and Pop_density. In this study, permutation importance was used to evaluate the contribution of environmental factors. The four most important variables were Dis_coastline (28.52%), BIO14 (6.76%), BIO4 (3.87%), and Pop_density (3.40%), were selected for further analysis and visualization. The potential suitable habitat for Milu was characterized by proximity to the coastline, driest-month precipitation of 30–40 mm, moderate annual temperature seasonality, and low human disturbance (Figure 3).

### 3.2. Potential Habitats of Milu Under Different Future Climate Scenarios

Potential suitable habitats for Milu were projected to expand in the central and northern coastal areas of the study region under future climate scenarios (Figure 2, Table 2), forming large and relatively continuous high suitability patches. In contrast, the southern region retained a coastal belt-like distribution pattern and was dominated by moderate and low suitability areas. According to the model predictions, under the SSP126 scenario for 2041–2060, the total suitable area increased to 72.2% of the study area, and the high-suitability area increased to 0.554 × 10^4^ km^2^. Under SSP245, the total suitable area reached 79.5%, with a high-suitability area of 0.554 × 10^4^ km^2^, indicating a substantial increase in the predicted total suitable area. In contrast, under SSP370, the total suitable area was relatively smaller, accounting for 57.9% of the study area, and the high-suitability area was only 0.187 × 10^4^ km^2^. By 2061–2080, the total suitable areas under SSP126, SSP245, and SSP370 accounted for 68.3%, 61.9%, and 60.5% of the study area, respectively, while the high-suitability areas ranged from 0.362 × 10^4^ to 0.577 × 10^4^ km^2^, indicating relatively small changes in the predicted area.

### 3.3. Driving Mechanisms of Environmental Factors Affecting the Distribution of Milu

#### 3.3.1. Interaction Detection Results

**Figure 4:**
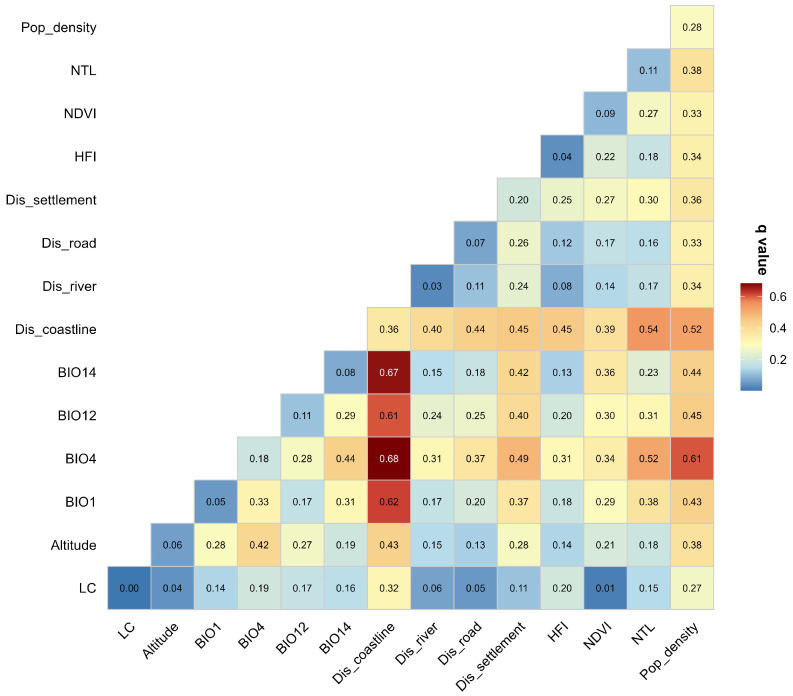
Interaction detector results for environmental factors affecting the spatial differentiation of potential habitat suitability for Milu. The values in the matrix represent the q-values of the interaction effects between pairs of environmental factors, and the color gradient from blue to red indicates increasing explanatory power. Higher q-values indicate stronger joint effects of two factors on the spatial pattern of potential habitat suitability.

Based on the factor detection results, all environmental factors except LC showed significant explanatory power (*p* < 0.01) [67]. The interaction detection results showed that the interactions between Dis_coastline and BIO4, and between Dis_coastline and BIO14, showed the strongest interaction effects, with q-values of 0.68 and 0.67, respectively (Figure 4). These results were consistent with the three most important environmental variables identified by the Biomod2 model. Both interactions exhibited nonlinear enhancement, indicating that coastline proximity and climatic factors jointly constrained the future distribution pattern of suitable habitat for Milu.

#### 3.3.2. Spatiotemporal Changes and Threshold Responses of Climatic Factors

Based on the spatial distribution pattern represented by distance to the coastline, BIO4 and BIO14 were identified as key climatic factors limiting the distribution of potential habitats for Milu. Based on the response curves in Figure 3, suitability threshold boundaries for BIO4 and BIO14 were mapped to analyze the spatial distribution of potentially suitable habitat (Figure 2). The results showed that the suitable threshold boundaries of BIO4 and BIO14 both shifted northward to varying degrees, indicating that changes in temperature seasonality and improved water availability during the driest month jointly influenced the adjustment of potential suitable area boundaries. In particular, under the SSP245 scenario during 2041–2060, the suitable ranges of BIO4 and BIO14 showed a high degree of spatial overlap, corresponding to a significant increase in the total suitable area. In some inland and southern areas, suitable climatic threshold conditions were not satisfied, thereby limiting the expansion of potential suitable habitats.

## 4. Discussion

### 4.1. Current Distribution of Potential Suitable Habitats for Milu

We found that currently suitable habitat for Milu was predicted to be distributed mainly along the coastline, showing a clear belt-like aggregation pattern. The high-suitability area is relatively small and is mostly concentrated in local areas with favorable ecological conditions. Previous studies have shown that suitable summer habitats for Milu in Shishou, Hubei Province, are mainly concentrated in mudflats, low grasslands, and bare land, and that occurrence probability and habitat suitability decrease with increasing distance from these habitat types [15]. During the low-water period of the Yangtze River, habitat selection is affected by water sources, human disturbance, and shelter conditions, with Milu tending to avoid disturbance and remain close to water sources [68]. Other studies have also indicated that potential habitats for Milu depend on wetlands, open vegetation, and water sources; these habitats exhibit patchy distributions in inland areas and belt-like aggregation along the coast and are therefore vulnerable to development activities [20]. The results of this study are generally consistent with the habitat-selection patterns reported for Milu in other regions. With the acceleration of coastal urban construction, transport infrastructure development, and land-use adjustment, coastal wetlands may continue to be occupied [69], further reducing suitable habitat areas. Therefore, moderate- and high-suitability areas should be designated as key conservation areas, and critical habitats such as coastal wetlands should be prioritized for protection and restoration.

### 4.2. Potential Habitats and Driving Factors Under Future Climate Scenarios

Under future climate change scenarios, the model projections indicate that potentially suitable habitat for Milu tends to expand in the central and northern coastal areas of Jiangsu and along the coastline. By contrast, the expansion of highly suitable habitat in the southern region appears to be relatively limited, probably because of stronger impacts of urbanization and intensive land use. According to the BAM framework, species distributions are jointly constrained by biotic interactions, habitat fragmentation, intraspecific competition, and movement or dispersal capacity [70,71]. Therefore, projections of future suitable areas should be interpreted with caution and further evaluated in relation to actual habitat conditions.

Both variable-importance and interaction-detection analyses showed that Dis_coastline, BIO14, BIO4, and Pop_density were the key factors shaping potential habitat suitability for Milu. Among these factors, BIO4 and BIO14 reflect key climatic conditions at the regional scale. Milu generally prefers wetland habitats with sufficient water availability, relatively abundant vegetation, and low levels of human disturbance. Temperature seasonality may affect habitat use across seasons by influencing vegetation phenology, food availability, and energetic costs of movement. Precipitation in the driest month reflects water availability during dry periods and may influence habitat selection by affecting water replenishment during the low-water season, soil moisture, and wetland vegetation growth. From the perspective of niche shifts, the northward movement of the suitable threshold boundaries of BIO4 and BIO14 under constraints related to coastal proximity suggests that the environmental space matching the core climatic niche of Milu may shift within coastal Jiangsu. Differences among emission scenarios in their effects on temperature seasonality and dry-season water availability may alter the suitable ranges and spatial overlap of BIO4 and BIO14, thereby influencing the extent of the northward shift and expansion of potential suitable habitat for Milu. Previous studies have shown that BIO4 and BIO14 are important climatic factors affecting the distribution of ungulates. Jia et al. [72] found that BIO14 contributed most to habitat suitability for *Cervus nippon sichuanicus*, indicating the key role of water availability during the driest period. Li et al. [73] reported that BIO4 was an important climatic factor affecting the distribution of *Cervus nippon kopschi*, reflecting its sensitivity to seasonal temperature variation. Zhu et al. [74] also found that BIO14 had a significant influence on some ungulate species in a multi-species study. The importance of BIO4 and BIO14 in this study is consistent with these findings, the results indicate that temperature seasonality and dry-season moisture conditions are key climatic factors driving future changes in potential suitable habitats for Milu. Therefore, conservation management should focus on nearshore wetlands and implement ecological restoration in areas sensitive to climatic fluctuations.

### 4.3. Conservation and Management Strategies for Milu Habitats

Future conservation and management of Milu should integrate spatial planning, population management, and technological monitoring to establish a multidimensional and coordinated strategy. First, in response to the projected northward shift in potentially suitable areas, cross-regional ecological corridors should be planned and habitat patch connectivity should be maintained. Second, to address local overpopulation and habitat degradation, measures such as ex situ conservation and rotational habitat restoration should be implemented to maintain a balance between population size and ecological carrying capacity [75]. Meanwhile, the expansion of coastal agriculture and aquaculture should be strictly controlled, buffer zones should be established, ecological compensation mechanisms should be improved, and wildlife-friendly traffic-isolation and road-crossing mitigation facilities should be optimized.

Overall, this study analyzed the spatial differentiation and future changes in potentially suitable habitat for Milu, but several limitations remain. The Biomod2 and OPGD models relied primarily on macroclimatic factors and static geographic conditions, with insufficient consideration of factors such as microtopography, water salinity [76,77], primary productivity, aquaculture, tidal inundation frequency, and the expansion of the invasive plant *Spartina alterniflora*. In addition, future land-use change and urban expansion were not incorporated. These factors may affect the actual use of coastal mudflats by Milu and the carrying capacity of their habitat. As the range of rewilded Milu expands, human-Milu conflict has become increasingly prominent; however, this study did not sufficiently examine behavioral responses, stress status, or fine-scale dispersal pathways. In addition, because the occurrence records used in this study were derived mainly from GPS collar tracking and were limited in data type, the model results should be interpreted primarily as reflecting potentially suitable habitat for the rewilded Milu population in coastal Jiangsu. Future studies should integrate multi-source remote sensing images from Sentinel and Landsat, hydrological and tidal dynamic data, field measurements of soil and water salinity, primary productivity indicators, remote-sensing-derived maps of *Spartina alterniflora*, urban expansion scenarios, and landscape fragmentation and connectivity indices. These data should be combined with field surveys, infrared cameras, Internet of Things technologies, and intelligent recognition techniques [78] to further develop more refined habitat suitability models for Milu and provide a stronger basis for population management and conflict mitigation.

## 5. Conclusions

Potentially suitable habitat for Milu in the coastal wetlands of Jiangsu is currently distributed mainly along the coastal zone and is projected to expand under future climate scenarios, particularly in the central and northern coastal areas. Coastline proximity, BIO14, BIO4, and population density are the key factors shaping habitat suitability. Future conservation should prioritize highly suitable habitats, maintain habitat connectivity, reduce human disturbance, strengthen wetland protection, and promote vegetation restoration.

## Figures and Tables

**Table 1:** Twenty-nine environmental variables and their descriptions.

Variable Abb.	Description	Unit	Primary Resolution
BIO1	Mean Annual Temperature	°C	1 km
BIO2	Mean Diurnal Temperature Range	°C	1 km
BIO3	Isothermality (BIO2/BIO7) (×100)	ratio	1 km
BIO4	Temperature Seasonality (standard deviation × 100)	°C	1 km
BIO5	Max Temperature of Warmest Month	°C	1 km
BIO6	Minimum Temperature of Coldest Month	°C	1 km
BIO7	Temperature Annual Range (BIO5-BIO6)	°C	1 km
BIO8	Mean Temperature of Wettest Quarter	°C	1 km
BIO9	Mean Temperature of Driest Quarter	°C	1 km
BIO10	Mean Temperature of Warmest Quarter	°C	1 km
BIO11	Mean Temperature of Coldest Quarter	°C	1 km
BIO12	Annual Precipitation	mm	1 km
BIO13	Precipitation of Wettest Month	mm	1 km
BIO14	Precipitation of Driest Month	mm	1 km
BIO15	Precipitation Seasonality (Coefficient of Variation)	1 cv	1 km
BIO16	Precipitation of Wettest Quarter	mm	1 km
BIO17	Precipitation of Driest Quarter	mm	1 km
BIO18	Precipitation of Warmest Quarter	mm	1 km
BIO19	Precipitation of Coldest Quarter	mm	1 km
Altitude	Altitude	m	30 m
Dis_coastline	Distance to Coastline	m	shp
Dis_river	Distance to the Nearest River	m	shp
Dis_road	Distance to the Nearest Road	m	shp
Dis_settlement	Distance to the Nearest Settlement	m	shp
HFI	The Human Footprint Index	Dimensionless index	1 km
LC	Land Cover	Categories 1–8	30 m
NDVI	Normalized Difference Vegetation Index	Dimensionless index	1 km
NTL	Nighttime Light	nW/cm^2^/sr	1 km
Pop_density	Population density	persons·km^−2^	1 km

**Table 2:** Statistics of suitable areas for Milu under different climate scenarios.

Year_Climate Scenario	HSA (10^4^ km^2^)	MSA (10^4^ km^2^)	LSA (10^4^ km^2^)	Total (%)
current	0.035	0.108	0.227	11.7
2041–2060_SSP126	0.554	0.889	0.834	72.2
2041–2060_SSP245	0.554	1.019	0.932	79.5
2041–2060_SSP370	0.187	0.762	0.875	57.9
2061–2080_SSP126	0.362	0.716	1.076	68.3
2061–2080_SSP245	0.577	0.786	0.588	61.9
2061–2080_SSP370	0.577	0.699	0.630	60.5

## Data Availability

The data presented in this study are available on request from the corresponding author.
